# National Growth Charts for the United Arab Emirates

**DOI:** 10.2188/jea.JE2008037

**Published:** 2008-12-17

**Authors:** Yousef M Abdulrazzaq, Mohamed A Moussa, Nicolaas Nagelkerke

**Affiliations:** 1 Department of Paediatrics, Faculty of Medicine and Health Sciences, UAE University, Al Ain, United Ara Emirates; 2 Department of Community Medicine, Faculty of Medicine and Health Sciences, UAE University, Al Ain, United Ara Emirates

**Keywords:** growth charts, UAE, height, weight, head circumference

## Abstract

**Background:**

Information on the health and growth status of the population is essential for planning and administering health promotion programs.

**Methods:**

This is a cross-sectional study of the anthropometric measurements of United Arab Emirates (UAE) children aged 0-18 years, by a multistage stratified random sampling technique based on age and sex. Healthy, full-term children of UAE nationality who did not have any diseases that could affect their growth pattern were included in the study. Children were selected using multistage sampling, using sampling proportional to size methods in 9 geographical areas. Growth charts for various anthropometric measures were created using Cole’s LMS statistical package. This package estimates age-specific percentiles with the use of smoothing splines after transformation to normality.

**Results:**

A total of 21,068 children (12,159 females) between the ages of 0 and 18 years were studied. In the present study, we included 8-15% of the population aged 0-18 years. The growth chart for 0-36 months is very similar to the NCHS growth reference chart in terms of both weight for age and length and height for age. The mean (+SD) length/height in children was 49.9 ± 3.2 cm at birth, 75.9 ± 5.7 cm at 12 months, 86.4 ± 4.5 cm at 24 months, 95.1 ± 5.9 cm at 36 months, and 111.1 ± 6.4 cm at 60 months. The height of UAE children in the first 3 years of life, especially at the ages of 2 and 3 years, mirrored those achieved by Brazilian children in the WHO study.

**Conclusion:**

The results of the present study are useful for growth assessment of UAE children.

## INTRODUCTION

Growth charts are essential tools used by health care workers, for assessing and monitoring growth in children. While international growth charts are available,^1^^,^^2^ they do not take into account variations in healthy growth among populations, which are attributable to genetic, environmental, nutritional, or other differences among populations. Some variations, such as variations in the age of onset of puberty (and the growth spurt that accompanies it) are poorly understood.^3^ Thus, the use of non-population-specific growth charts may have various consequences, for instance, the misclassification of otherwise perfectly healthy and developing children as underweight. There is a lack of national growth charts in the United Arab Emirates (UAE), compelling UAE clinicians to use World Health Organization, British, or American growth charts. This may not be the ideal reference for assessing UAE children since normal, healthy children in the UAE may follow a somewhat different growth pattern. In the UK, Asian babies were found to be, on an average, lighter than European babies.^4^ However, Asians in Britain are not a homogenous group, as a later study^5^ found that the growth of Sikhs at 3 years of age was, on an average, comparable to that of Europeans at the same age. Hindus had the lowest weight, while Muslims had intermediate weight. Rona and Chinn^6^ found that, on an average, Afro-Caribbean children were approximately 3.5 cm taller and Gujarati-speaking children were approximately 3 cm shorter than white children on an average.

The National Centre for Health Statistics (NCHS) has conducted a large survey of the growth characteristics of children in the USA. These children represented a cross-section of different ethnic and economic groups and are regarded as reference standards for the USA.^7^ Data were collected from measurements taken as a part of a series of national health examination surveys conducted by the NCHS from 1964 to 1994. New standard growth charts were constructed with the data obtained, and like the previous editions, these charts could be used as international standards. A newer version—the 2000 Center for Disease Control and Prevention (CDC) growth charts—has been constructed, and is a revised and updated version of the 1997 growth chart.

In the UK, the Tanner-Whitehouse Charts have been used as the standard, but due to concerns related to secular trends toward earlier maturity and adult height, new standards have recently been published.^8^^-^^10^ Some countries in the Gulf Cooperation Council have previously established growth charts for their population.^11^^-^^13^ Recently, WHO published growth charts, which represented the data from a collaboration between a diverse set of countries: Brazil, Ghana, India, Norway, Oman, and the USA, which came together to participate in the WHO Multicentre Growth Reference Study.^1^ The UAE Multicentre Growth Study (UAEMCGS) was therefore undertaken to provide comprehensive data on the growth pattern of UAE children aged 0-18 years, and to compare these patterns with the NCHS/CDC/WHO international reference values.

## SUBJECTS AND METHODS

The UAE consists of a federation of 7 princely states (emirates) that differ in size and population. In 1992, the UAE had an overall population of 2,011,400 (1992 Annual Report), 400,000 of whom were estimated to be nationals. In the study year 1991-92, the number of children under 5 years of age was estimated to be 57,640 on the basis of projections from the 1989 data, assuming an annual growth rate of 4.6% (Annual Statistics Book, Table 1).

**Table 1:** Age-sex distribution of the children who were studied

Age In Years	Sex		Total
	
	Female	Male	0.00
.00	583	602	1,185
1.00	168	186	354
2.00	85	56	141
3.00	72	85	157
4.00	588	524	1,112
5.00	881	662	1,543
6.00	631	478	1,109
7.00	647	527	1,174
8.00	745	567	1,312
9.00	730	564	1,294
10.00	708	589	1,297
11.00	730	615	1,345
12.00	809	510	1,319
13.00	732	631	1,363
14.00	742	568	1,310
15.00	841	489	1,330
16.00	877	530	1,407
17.00	732	336	1,068
18.00	339	143	482
19.00	128	14	142
20.00	49	1	50
Total	11,817	8,677	20,494

Children were sampled as follows:

### I. Pre-school Children Aged 0-5 years Sampling Technique

A national cross-sectional growth survey of preschool-age children was conducted using multistage stratified random sampling. It surveyed 0-60 month old children, specifically at the ages of 0 (birth, data obtained from hospitals), 0.5 to <1.5, ≥1.5 to <2.5, ≥2.5 to <3.5, ≥5.5 to <6.5, ≥8.5 to <9.5, ≥11.5 to <12.5, ≥17.5 to <18.5 (defined by the number of times the children received scheduled vaccinations), ≥23 to <25, ≥29 to <31, ≥35 to <37, ≥41 to <43, ≥47 to <49, ≥53 to <55, and ≥59 to <61 months (from nurseries and kindergartens).^9^ The sample size (10.2% of the population) was determined with the objective to include at least 200 children in each age-sex group, which provides sufficient precision for the construction of growth charts.

The sampling procedure was as follows:

1. The country was divided into 9 districts (strata): Abu Dhabi, Al-Ain, Dubai, Sharjah, Ajman, Umm-Al-Quwain, Ras Al-Khaimah, Fujairah, and the western sector of Abu Dhabi.
  A sample of districts was taken from the 9 described above (primary sampling units), and sampled with a probability proportional to (population) size.
2. Nurseries, kindergartens, and immunization centers in each selected district were enumerated and stratified according to the sex and age of the children.
3. Nurseries, kindergartens, and immunization centers were then randomly drawn from each substratum (second-stage sampling) and enumerated.

A simple random sample, proportional to size, was then drawn from each unit (third-stage sampling).

### Inclusion and Exclusion Criteria

Healthy, full-term children of UAE nationality (born to a father of Emirates nationality) who had no diseases that could affect their growth pattern were included in the study. Children who suffered from diseases such as anemia other than iron-deficiency anemia and diabetes mellitus and had congenital malformations and debilitating chronic diseases like asthma were excluded. Moreover, children whose mothers had diseases that could possibly affect the growth pattern of their children during the neonatal period (e.g., diabetes mellitus) were excluded.

### Methods of Measurement

Age was determined to the nearest month using the date of birth ascertained from the birth certificates until the date of measurement. Length or height was measured to the nearest 0.1 cm with a Nivotoise portable height gauge. Weight was measured to the nearest 0.1 kg with the use of a scale with non-detachable weights. The methods of measurement were those recommended by Abdulrazzaq et al.^14^ Research assistants were trained to perform the measurements by one of the authors (YMA).

### II. School Children Aged 6-18 years Sampling Technique

Information on the school children registered for the school year 1991/92, distributed according to sex, geographical area, and educational level, were obtained from the Ministry of Education. On the basis of the available facilities such as trained staff, transportation, and funding and feasibility, we decided to include 15% of the total school children (104,182 children) registered for that school year. An attempt was made to include not less than 500 children of each sex per annual age group. Age was validated from school records, which in turn were based on birth certificates.

First, schools in all 9 districts were sampled proportional to size. In each district, schools were enumerated and randomly selected using random tables. Then, children were selected from each school by using school records according to a stratified random sampling technique. Only Emirati children (i.e., UAE nationals) were included in the survey. A replacement sample was selected in each school to substitute any dropouts. The age intervals were ≥5.5 to <6.5, ≥6.5 to <7.5, ≥7.5 to <8.5, ≥8.5 to <9.5, ≥9.5 to <10.5, ≥10.5 to <11.5, ≥11.5 to <12.5, ≥12.5 to <13.5, ≥13.5 to <14.5, ≥14.5 to <15.5, ≥15.5 to <16.5, ≥16.5 to <17.5, ≥17.5 to <18.5, and ≥18.5 to <19.5.

### Methods of Measurement

The survey team included 18 trained female paramedical assistants, 2 per region. The assistants measured weight, height, head circumference, and mid-arm circumference independently (one for each sex). Height was measured by 2 persons (one for each sex). In order to minimize intra-observer error, only 2 persons measured the head circumference.

Weight was measured to the nearest 0.1 kg using a Detecto scale with a 140-kg capacity (Detecto Scales Inc., Brooklyn, N.Y). Weight measurements were taken without shoes and with as few clothes on as possible. Due to cultural obligations, weight was measured in school uniforms (light shirt and trousers for boys and light blouse and dress for girls). To minimize errors in measurements, the weighing scales were checked before each session to ensure that the unloaded scale registered zero. The weighing scale was also frequently checked by weighing an object with known weight.

In order to obtain the nude weights of children, a sample of 130 children (65 boys and 65 girls) was randomly drawn. The children were divided into 1-year age groups, and then 10 children were selected randomly from each age group (5 for each sex). The weights of the children with and without clothes were obtained, and the weight of the clothes was calculated. The average weight of clothes was then calculated for boys and girls.

Height was measured as the distance between 2 flat surfaces, using a stadiometer attached to the weighing scale. Standing height was measured to the nearest 0.1 cm. Each child stood erect and barefoot, with his/her heels, buttocks, and back touching the stadiometer. Then, the horizontal indicator of the stadiometer was lowered until it firmly touched the crown of the head.^14^

### Data Analysis

Incomplete or unclear forms and “impossible” outliers were excluded.

Anthropometric data were then analyzed using the LMS and SPSS statistical packages.

Growth charts for different weight, height, and head circumference anthropometric measurement, specifically their estimated 3^rd^, 10^th^, 25^th^, 50^th^, 75^th^, 90^th^, and 97^th^ age-sex specific percentiles, were created using the LMS statistical package.^15^^,^^16^ The LMS method uses a Box-Cox power transformation (with power *L*), which normalizes the data at any given age. At any age, the distribution is characterized by *L* as well as by *M* (the generalized mean of the data) and *S* (the generalized coefficient of variation). Splines were used for smoothing these values across different ages. The degree of smoothing was decided on the basis of a visual inspection of the estimated percentile curves. Generally, the most flexible smoothing (i.e., using splines with the highest possible degrees of freedom) that did not show unrealistic jitter and non-monotonicities was chosen. In the case of height, the age groups 0-20 and 0-4 were fitted separately in order to accommodate the narrow distribution of lengths at birth. The results of the 0-4 fits were then used to replace the first 4 years of the 0-20 fit. The estimated percentile curves were exported to Microsoft Excel spreadsheets, and percentile graphs were constructed for the age group 0-18 years.

## RESULTS

**Figure 1.**
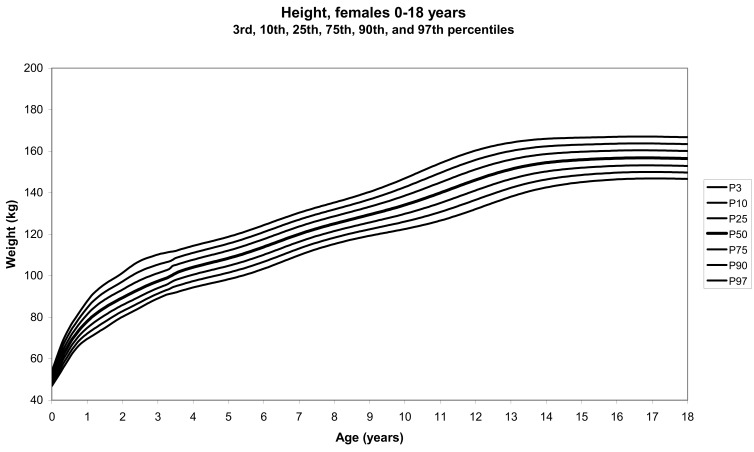
Line chart of height with age in female UAE children aged 0-18 years showing heights at the 3^rd^, 10^th^, 25^th^, 50^th^, 75^th^, 90^th^, and 97^th^ centiles at different ages

**Figure 2.**
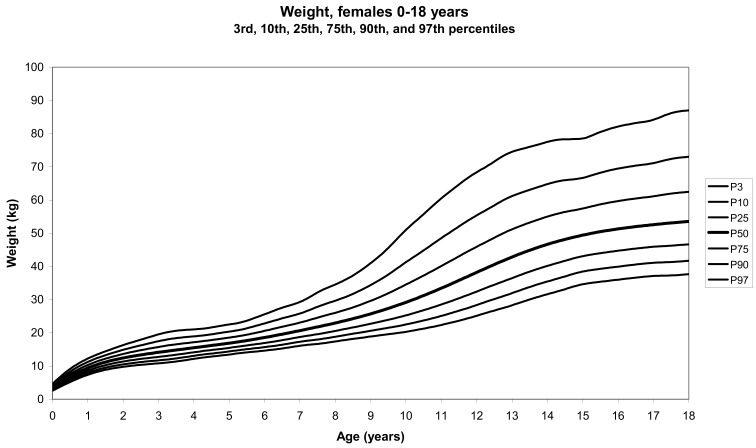
Line chart of weight with age in female UAE children aged 0-18 years showing weights at the 3^rd^, 10^th^, 25^th^, 50^th^, 75^th^, 90^th^, and 9^th^ centiles at different ages

**Figure 3.**
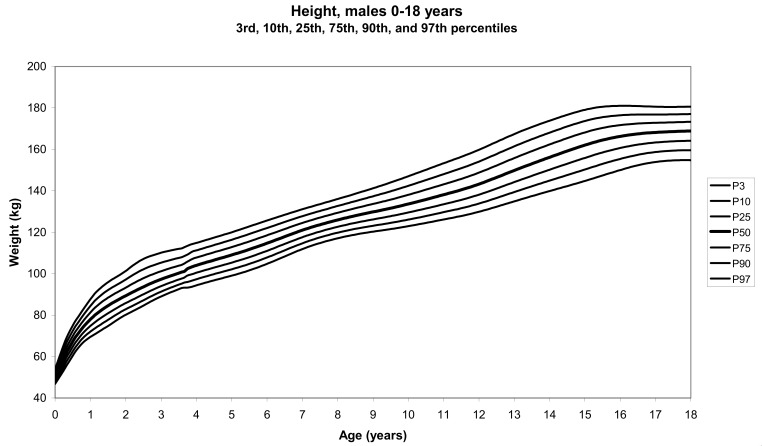
Line chart of height with age in male UAE children aged 0-18 years showing heights at the 3^rd^, 10^th^, 25^th^, 50^th^, 75^th^, 90^th^, and 95^th^ centiles at different ages

**Figure 4.**
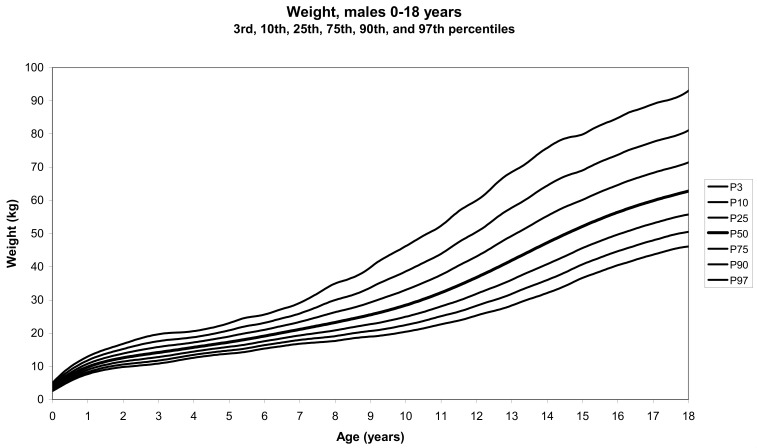
Line chart of weight with age in male UAE children aged 0-18 years showing weights at the 3^rd^, 10^th^, 25^th^, 50^th^, 75^th^, 90^th^, and 97^th^ centiles at different ages

**Figure 5.**
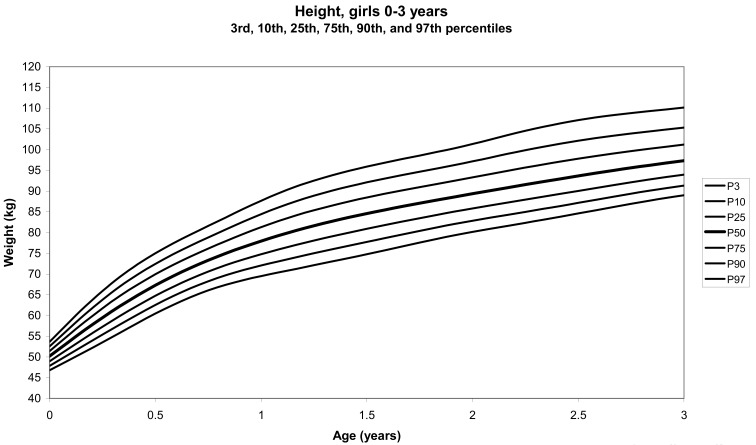
Line chart of length with age of girls aged 0-3 years showing lengths at the 3^rd^, 10^th^, 25^th^, 50^th^, 75^th^, 90^th^, and 97^th^ centiles at different ages

**Figure 6.**
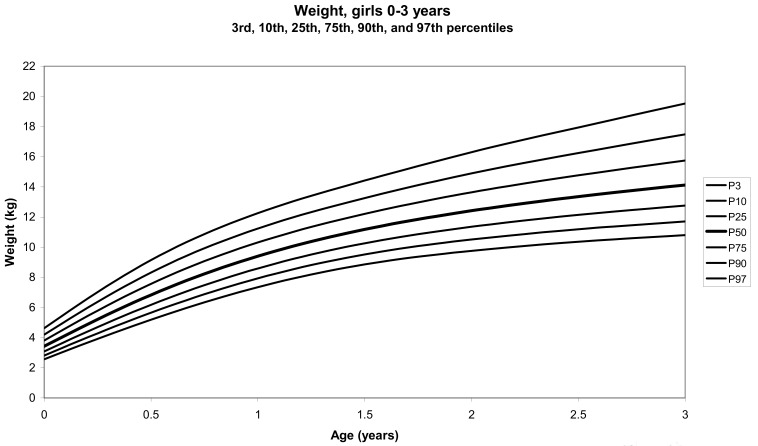
Line chart of weight with age of girls aged 0-3 years showing weights at the 3^rd^, 10^th^, 25^th^, 50^th^, 75^th^, 90^th^, and 97^th^ centiles at different ages

**Figure 7.**
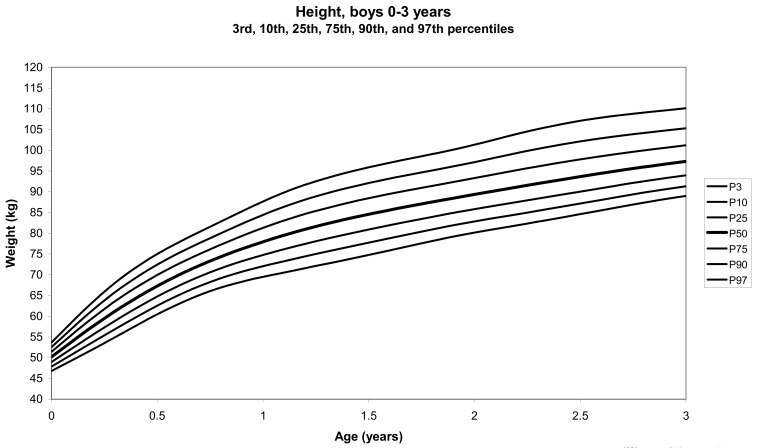
Line chart of length with age of boys aged 0-3 years showing lengths at the 3^rd^, 10^th^, 25^th^, 50^th^, 75^th^, 90^th^, and 97^th^ centiles at different ages

**Figure 8.**
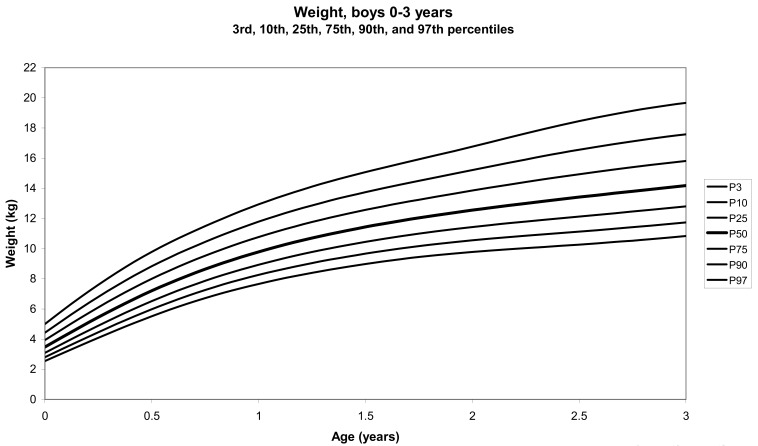
Line chart of weight with age of boys aged 0-3 years showing weights at the 3^rd^, 10^th^, 25^th^, 50^th^, 75^th^, 90^th^, and 97^th^ centiles at different ages

**Figure 9.**
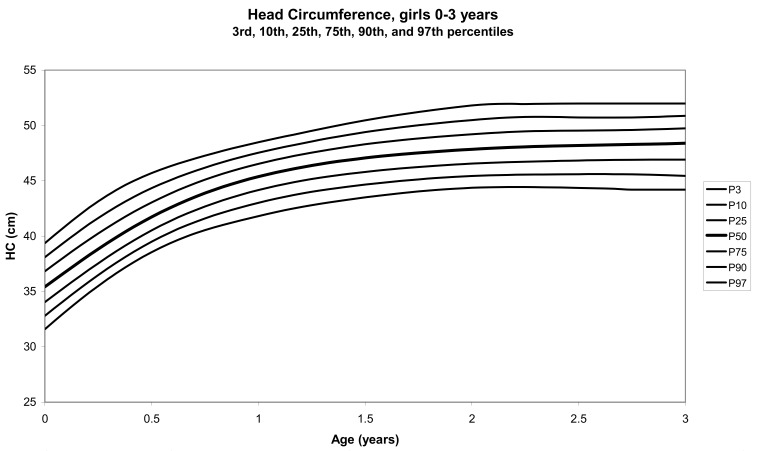
Line chart of head circumferences with age of girls aged 0-3 years showing head circumferences at the 3^rd^, 10^th^, 25^th^, 50^th^, 75^th^, 90^th^, and 97^th^ centiles at different ages

**Figure 10.**
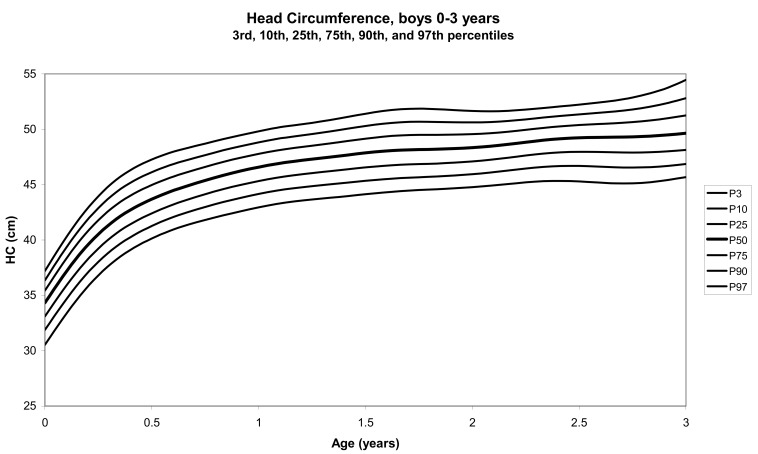
Line chart of head circumferences with age of boys aged 0-36 months showing head circumferences at the 3^rd^, 10^th^, 25^th^, 50^th^, 75^th^, 90^th^, and 97^th^ centiles at different ages

The total number of children approached was 21,118, among whom 50 were excluded because they did not meet the inclusion criteria. A total of 21,068 children (12,159 females) remained, and after data cleaning and exclusion of children over 20 years of age, 20,494 children were available for analysis. Table 1 shows the age-sex distribution of these subjects. Figures 1-4 show the height-for-age and weight-forage reference percentiles for boys and girls aged 0-18 years, and figures 5-10 show the reference percentile charts for height-age, weight-age, and head circumference-age for boys and girls aged 0-3 years. Each figure shows the 3^rd^, 10^th^, 25^th^, 50^th^, 75^th^, 90^th^, and 97^th^ percentiles corresponding to SD scores of -1.88 to +1.88 after transformation to normality.

The mean length, weight, and head circumference for a female newborn baby were 52.2 cm, 3.4 kg, and 35.4 cm, respectively; for a male baby, these values were 51.1 cm, 3.5 kg, and 34.3 cm, respectively. At 6 months, the mean length, weight, and head circumference for females were 62.0, 6.8, and 43.7 cm, respectively; for males, they were 63.2 cm, 7.2 kg, and 41.9 cm.

Table 2 shows a comparison of growth patterns in the UAE to international standards based on different populations.^11^ Children in the UAE are comparable to other populations from birth to 3 years of age, but on an average, are smaller at 4 and 5 years of age.

**Table 2:** Comparisons between the means of lengths/heights of children from 0-5 years from different countries^1^

Age (months)	UAE	Brazil	Ghana	Oman	USA	India	Norway
0	49.9 ± 3.15	49.6 ± 1.9	49.5 ± 1.9	49.2 ± 1.7	49.7 ± 2.0	49.0 ± 1.8	50.4 ± 1.9
6	64.9 ± 7.7	66.8 ± 2.4	66.5 ± 2.3	66.1 ± 2.0	66.3 ± 2.4	66.6 ± 2.3	67.9 ± 2.4
12	75.9 ± 5.7	75.4 ± 2.7	75.2 ± 2.7	74.4 ± 2.4	74.5 ± 2.7	75.0 ± 2.3	75.5 ± 2.4
18	81.6 ± 5.6	82.4 ± 3.0	82.0 ± 2.8	80.9 ± 2.7	81.7 ± 3.0	81.5 ± 2.9	82.1 ± 2.8
24	87.5 ± 4.5	88.4 ± 3.2	87.5 ± 3.0	86.4 ± 3.1	87.4 ± 3.3	87.0 ± 3.2	87.8 ± 3.1
24-26	89.9 ± 3.8	88.9 ± 3.0	87.1 ± 3.1	86.6 ± 3.7	-	87.0 ± 4.0	87.3 ± 3.4
36-38	97.4 ± 5.9	97.9 ± 4.0	96.3 ± 4.0	95.3 ± 3.8	95.9 ± 3.9	95.4 ± 4.3	96.7 ± 3.6
48-50	103.9 ± 9.0	104.9 ± 4.5	104.3 ± 4.5	101.8 ± 4.3	103.3 ± 3.5	103.3 ± 3.8	103.6 ± 3.7
60-62	108.6 ± 6.4	111.2 ± 5.0	112.6 ± 6.0	109.00 ± 4.1	109.6 ± 4.8	108.8 ± 3.6	110.6 ± 4.2

## DISCUSSION

Presented in this paper are the first growth charts for UAE children, developed using a representative sample of both breast- and formula-fed infants, as well as of children of school-going age. Since almost all Emirati children attend primary and secondary schools, our sample is highly representative of the total healthy population, although we may have overlooked children who were temporarily absent due to minor illnesses. The growth chart for 0-36 months is very similar to the CDC growth reference charts both in terms of weight for age and length and height for age. This similarity in growth during infancy seems consistent with comparisons made among diverse populations, which report considerable homogeneity.^17^^,^^18^ In comparison to the countries involved in the WHO Child Growth Standards (WHO CGS) study (USA, Brazil, Norway, India, Oman, and Ghana), the heights in the first 3 years of life, notably at 2 and 3 years of age, apparently specifically resembled those achieved by Brazilian children (Table 2).^1^ However, there were also differences especially at 6 months and 60 months of age: UAE children were significantly smaller than similar-aged children from other countries in the WHO study. The comparisons of adult (18-year-olds) height with that of other countries show more striking differences. As expected, the heights of young UAE adults exceed those in developing countries such as India,^19^ where even in Punjab, the state with the tallest men, young adult men and female only reach an average height of 168.6 and 154.6 cm, respectively; this is clearly less than the corresponding average heights in the UAE, which are 173.4 and 156.4 cm. Many other states in India fall far short even of the figures in Punjab; this difference could be due to genetic reasons, but it is more likely due to poor nutrition and health care. Nevertheless, during the entire period these children grew up, the UAE was a wealthy country with adequate health care and abundant food; therefore, one might expect the adult heights to match those in the USA. However, this is apparently not the case, since the median height of 18-year-old men and women in the USA is 176.0 and 163.6 cm, respectively.^20^

The head circumferences of neither males nor females in the present study were significantly different in any of the age groups, compared to the corresponding figures in the USA.^20^ The increase in head circumference with age has shown a positive secular trend both in the USA and Japan.^21^ In Japan, although the head circumferences of children below 6 years of age were smaller than those of Caucasian children, the head circumference/height ratios were significantly higher in all age groups and were especially prominent after puberty.^21^ In the present study, there were no significant differences in the median head circumference between UAE and American children under 3 years of age. This was expected since only minor differences existed in the other growth parameters. There was also no significant difference with regard to the measurements in children less than 3 years of age from different countries in the WHO Multicentre Study.

Our charts were constructed using the LMS method, which is also used in the construction of the CDC growth charts. The LMS program is very flexible, since the degrees of smoothing can be set by the user, and has been widely used to identify both normal and extreme growth patterns.^15^ Unfortunately, our charts only apply to UAE nationals. A majority of the UAE population comprises expatriates from more than 120 different countries of origin. With this vast number of disparate nationalities, it would have been difficult to develop standards that would apply to children from every nationality; we have therefore limited our study to UAE nationals. Another limitation of our study, as well as the growth curves in general, is that they are cross-sectional. With some exceptions (e.g., the WHO CGS study for children under 2 years of age), most growth charts are also constructed from cross-sectional data. To assess the growth paths of individual children, measurements taken at different ages are often available. The interpretation of a growth path that follows approximately the 50^th^ percentile for height for 3 years and then drops quickly to the 3^rd^ percentile should differ from that of one that follows the 3^rd^ percentile consistently. However, our growth curves provide no indication as to how this can be achieved. Alternative approaches have therefore been developed. For example, Karlberg^22^ presented the infancy-childhood-puberty model in which he constructed 3 charts for the 3 periods and then combined them to form the Covenant Care Pediatrics (CCP, http://www.drpulliam.net/prc/updates/newgrowth.aspx) standard for growth. This approach still needs to be applied to the UAE population.

In some respects, our choices were different from those of the recently published WHO Multicentre Growth Reference Study.^1^ Unlike this study, which aimed at setting norms, we did not restrict ourselves to breast-fed children but documented the growth of apparently healthy children in the UAE. In the case of a child with an extreme value (e.g., a low percentile for height), it should not be considered a violation of “norms” but rather as a sign of something being amiss.

The approaches to growth curve development appear to differ roughly along the descriptive-normative dimension (although all growth curves constitute a mixture of the two). One’s position along this dimension determines whether only breast-fed children or all children should be included. This is important as, generally, breast-fed infants grow more rapidly in the first 2 months of life and not as rapidly at 3-4 months.^23^ Similarly, it has an impact on whether population-specific or uniform standards should be used. The new CDC reference represents the combined growth patterns of both breast- and formula-fed infants in the USA. Breast-fed infants comprised half the infants born, and about a third of those were breast-fed for 3 months or longer. CDC and the WHO promote one set of growth charts for all racial and ethnic groups as differences in growth among various racial and ethnic groups are perceived to be the result of environmental^24^ rather than genetic influences. The study by Mei et al^24^ shows the trend of the prevalence of low height-for-age or stunting in recently immigrated refugee children from Southeast Asia to the USA in the early 1980s, and compares it to that of white children living in the USA. By the 1990s, the prevalence of low height-for-age had declined among Asian children, and height-forage was almost identical to that of white children in the USA. They concluded that improved growth was due to the changing socioeconomic status. That this increase in growth truly represented an improvement in that it leads to longer and healthier lives has remained an assumption that has been challenged empirically.^25^

In conclusion, we provide, for the first time, growth curves of children in the UAE, which show few differences in comparison with the WHO reference charts. UAE children in the age range of 4-5 years are smaller than their counterparts in the reference charts. The young adult heights with regard to both males and females are lower in UAE nationals than in US young adults of the same age.
